# Yoga Practice Is Beneficial for Maintaining Healthy Lifestyle and Endurance Under Restrictions and Stress Imposed by Lockdown During COVID-19 Pandemic

**DOI:** 10.3389/fpsyt.2021.613762

**Published:** 2021-06-22

**Authors:** Raghuram Nagarathna, Akshay Anand, Manjari Rain, Vinod Srivastava, Madhava Sai Sivapuram, Ravi Kulkarni, Judu Ilavarasu, Manjunath N. K. Sharma, Amit Singh, Hongasandra Ramarao Nagendra

**Affiliations:** ^1^Divison of Life Sciences, Swami Vivekananda Yoga Anusandhana Samsthana, Bengaluru, India; ^2^Department of Neurology, Post Graduate Institute of Medical Education and Research, Chandigarh, India; ^3^Centre for Mind Body Medicine, Post Graduate Institute of Medical Education and Research, Chandigarh, India; ^4^Centre of Phenomenology and Cognitive Sciences, Panjab University, Chandigarh, India; ^5^College of Health and Behavioral Sciences, Fort Hays State University, Hays, KS, United States; ^6^Department of General Medicine, Dr. Pinnamaneni Siddhartha Institute of Medical Sciences and Research Foundation, Chinna-Avutapalli, India; ^7^Division of Yoga and Physical Sciences, Swami Vivekananda Yoga Anusandhana Samsthana, Bengaluru, India

**Keywords:** COVID-19, Yoga, global health, stress, coping straregies

## Abstract

Uncertainty about Coronavirus disease 2019 (COVID-19) and resulting lockdown caused widespread panic, stress, and anxiety. Yoga is a known practice that reduces stress and anxiety and may enhance immunity. This study aimed to (1) investigate that including Yoga in daily routine is beneficial for physical and mental health, and (2) to evaluate lifestyle of Yoga practitioners that may be instrumental in coping with stress associated with lockdown. This is a pan-India cross-sectional survey study, which was conducted during the lockdown. A self-rated scale, COVID Health Assessment Scale (CHAS), was designed by 11 experts in 3 Delphi rounds (Content valid ratio = 0.85) to evaluate the physical health, mental health, lifestyle, and coping skills of the individuals. The survey was made available digitally using Google forms and collected 23,760 CHAS responses. There were 23,290 valid responses (98%). After the study's inclusion and exclusion criteria of yogic practices, the respondents were categorized into the Yoga (*n* = 9,840) and Non-Yoga (*n* = 3,377) groups, who actively practiced Yoga during the lockdown in India. The statistical analyses were performed running logistic and multinomial regression and calculating odds ratio estimation using R software version 4.0.0. The non-Yoga group was more likely to use substances and unhealthy food and less likely to have good quality sleep. Yoga practitioners reported good physical ability and endurance. Yoga group also showed less anxiety, stress, fear, and having better coping strategies than the non-Yoga group. The Yoga group displayed striking and superior ability to cope with stress and anxiety associated with lockdown and COVID-19. In the Yoga group, participants performing meditation reportedly had relatively better mental health. Yoga may lead to risk reduction of COVID-19 by decreasing stress and improving immunity if specific yoga protocols are implemented through a global public health initiative.

## Introduction

WHO declared Coronavirus disease 2019 (COVID-19), originating from Wuhan, China, caused by Severe Acute Respiratory Syndrome Coronavirus-2 (SARS CoV-2), as a pandemic on March 11, 2020. To prevent spread and provide sufficient time for hospitals' readiness, the Governments worldwide had to impose “Lockdown” in their respective countries. Under lockdown, people were restricted from remaining outdoors with certain exceptions resulting from emergencies.

India imposed the world's most extensive lockdown on March 23, 2020 (1). Many people were either stranded in their homes or containment zones, disrupting small businesses' earnings, working of domestic maids, daily wagers, and laborers. In addition, the uncertainty of the disease's contagious nature among the public and healthcare workers led to fear, panic, anxiety, and stress. Stress also intensified among those with chronic illnesses, as susceptibility and severity of COVID-19 were associated with co-morbidities (2–4). Furthermore, global infodemic and fake news exasperated anxiety and stress among the general public (5, 6).

Previous studies have evidenced increased post-traumatic stress disorder (PTSD) after epidemic or natural calamities such as SARS, earthquake, or a tornado, including COVID-19 (7–10). Wang et al. conducted a comprehensive self-administered online survey in China to understand the prevalence of psychological stress in the COVID-19 pandemic. They reported increased panic, stress, anxiety, and depression similar to previous studies conducted during the 2003 SARS epidemic (7, 8, 11). A similar online survey by Liu et al. reported that 20% of people showed anxiety, 27% reported depression, 7.7% had psychological distress, and 10% suffered from phobias (12). Furthermore, there were changes in people's behavioral patterns due to lockdown, especially concerning their eating habits. Increased consumption of junk food, soft drinks, and alcohol resulted in obesity. Lockdown disrupted the daily routines, sleep hours, outdoor activities, and increased screen time and smoking, predisposing people to risks of COVID-19 (13, 14). Two small studies from India have shown similar trends (15, 16).

In the current study on the COVID-19 pandemic, it has been reported that the impact on psychological stress might be more pronounced due to persistent global media feeds and internet access. The present COVID Health Assessment Scale (CHAS) survey was designed to evaluate the physical and mental health and coping skills of participants who practiced yoga and those who did not. Several studies have shown that Yoga brings a positive change in physical and mental health by regulating the hypothalamic–pituitary–adrenal system, sympathetic nervous system, reducing the cortisol, and improving immunity indicated by an increase in CD4, heart rate, fasting blood glucose, cholesterol, and low-density lipoprotein levels (17–20). Thus, it appears that Yoga practitioners have healthy lifestyle among the general population. This study investigated that including Yoga in daily routine is beneficial for physical and mental health. Also, Yoga practitioners have a healthier lifestyle, which improves their ability to cope with the restrictions and stress under lockdown.

## Materials and Methods

The current study received ethical approval from Swami Vivekananda Yoga Anusandhana Samsthana (S-VYASA) University, Karnataka, India. CHAS, a unique self-assessment scale, was designed for the survey in 10 different languages, English, Hindi, Assamese, Bengali, Kannada, Malayalam, Marathi, Odia, Tamil, and Telugu. A committee consisting of 11 experts was constituted who undertook three rounds of discussions as per Delphi protocol and agreed to the CHAS questionnaire that assessed the positive and negative aspects of physical and mental health, lifestyle, and associated coping methods during the lockdown period (Table 1). Among 11 experts, 6 had PhD in Yoga with more than 15 years of experience in yoga research, 3 were post-graduate in yoga with experience of more than 10 years in yoga, one is a professor of statistics, a mathematician with masters in psychology and PhD in yoga, and one is a psychologist with PhD in yoga. Content valid ratio (CVR) was 0.85 for CHAS as per Delphi method (21–23). CHAS also collected the demographic (questions 1–10) and lifestyle (questions 39–64) details of the participants.

**Table 1:** CHAS questionnaire.

**Parameters**	**Question number**	**Questions**	**Valid responses**
Demographics	1	Gender	Female, male, other
	2	Age	<20 years, 20–30 years, 31–40 years, 41–50 years, 51–60 years, 61–70 years, 71–80 years, above 80 years
	3	Weight (kg)	Open-ended question
	4	Height (cm)	Open-ended question
	5	State	Not in India, Andhra Pradesh, Arunachal Pradesh, Assam, Bihar, Chhattisgarh, Goa, Gujarat, Haryana, Himachal Pradesh, Jharkhand, Karnataka, Kerala, Madhya Pradesh, Maharashtra, Manipur, Meghalaya, Mizoram, Nagaland, Odisha, Punjab, Rajasthan, Sikkim, Tamil Nadu, Telangana, Tripura, Uttar Pradesh, Uttarakhand, West Bengal, Andaman and Nicobar, Chandigarh, Delhi NCT, Dadra and Daman, Jammu and Kashmir, Ladakh, Lakshadweep, Puducherry
	6	Country	India, China, USA, Italy, UK, Spain, France, Other
	7	Occupation	Agriculture, business, employed, homemaker, retired, student, professional, other
	8	Education	Less than graduation, graduate, post-graduate
	9	During lock down staying with:	Family, friends, colleagues, alone, away from home
	10	During lock down are you:	Working from home, working from office, not working
COVID-19 exposure	11	Are you experiencing any of the following?	No symptoms, cough, fever, breathing difficulty, other
	12	Have you traveled anywhere internationally since January 2020?	Yes, no
	13	Which of the following apply to you?	Other, recent COVID-19 interacted, was quarantined, in quarantine, health worker, hospitalized
	14	Number of days passed since you interacted or lived with someone who has been tested positive for COVID-19	Open-ended question
	15	Number of days passed since you are in quarantine. Please ignore if you were not quarantined	Open-ended question
Physical health	16	How do you rate your physical strength and endurance?	Very good, good, average, bad, very bad
	17	History of chronic health problems	Healthy, BP, lung disease, heart disease, cancer, arthritis, diabetes, others
Mental health	18	Do you feel you are low in energy and downhearted during this lock-down period?	Not at all, somewhat, very much
	19	How anxious are you about the implications of COVID-19 in your life?	Not at all, somewhat, very much
	20	How much do the following issues worry you during this lock-down period? (Fear of getting infected and the associated physical suffering)	Not at all, somewhat, very much
	21	How much do the following issues worry you during this lock-down period? (Fear of death)	Not at all, somewhat, very much
	22	How much do the following issues worry you during this lock-down period? (Fear of a possible financial burden)	Not at all, somewhat, very much
	23	How much do the following issues worry you during this lock-down period? (Fear of unknown related to COVID-19)	Not at all, somewhat, very much
	24	How much do the following issues worry you during this lock-down period? (Fear of spreading infection to near and dear ones)	Not at all, somewhat, very much
	25	How do you rate your personality? (Are you generally goal driven; perfectionist and persistent)	Disagree, maybe, agree
	26	How do you rate your personality? (Are you caring and ready to help others all the time)	Disagree, maybe, agree
	27	How do you rate your personality? (Do you always feel insecure; stressed and have mood swings)	Disagree, maybe, agree
	28	How do you rate your personality? (Are you always open to new ideas and suggestions and willing to try them)	Disagree, maybe, agree
	29	How do you rate your personality? (Do you always enjoy sharing your thoughts and ideas with others)	Disagree, maybe, agree
Coping strategy	30	How do you rate your coping abilities during this lock-down period?	Poor, average, very good, excellent
	31	How do you prefer spending time (apart from your regular, work-related engagements) during this national lock-down period? (Watching TV/playing computer games)	Yes, no
	32	How do you prefer spending time (apart from your regular, work-related engagements) during this national lock-down period? (Reading/writing)	Yes, no
	33	How do you prefer spending time (apart from your regular, work-related engagements) during this national lock-down period? (Cooking)	Yes, no
	34	How do you prefer spending time (apart from your regular, work-related engagements) during this national lock-down period? (Exercise)	Yes, no
	35	How do you prefer spending time (apart from your regular, work-related engagements) during this national lock-down period? (*Yogasana*)	Yes, no
	36	How do you prefer spending time (apart from your regular, work-related engagements) during this national lock-down period? (Meditation)	Yes, no
	37	How do you prefer spending time (apart from your regular, work-related engagements) during this national lock-down period? (Faith-based practices including prayer etc.)	Yes, no
	38	How do you prefer spending time (apart from your regular, work-related engagements) during this national lock-down period? (Social media and Internet)	Yes, no
Lifestyle	39	As a coping strategy do you use? (Tobacco)	Never, occasionally, regularly
	40	As a coping strategy do you use? (Drink Alcohol)	Never, occasionally, regularly
	41	As a coping strategy do you use? (or use any other substance)	Never, occasionally, regularly
	42	Has the lock-down increased your dependency on use of tobacco, alcohol, or any other substances?	Yes, no, not applicable
	43	In general, how do you describe your eating habits as? (I am disciplined with respect to time and place of eating)	Yes, no
	44	In general, how do you describe your eating habits as? (I am a strict vegetarian/vegan)	Yes, no
	45	In general, how do you describe your eating habits as? (I like eating junk food)	Yes, no
	46	In general, how do you describe your eating habits as? (I like spicy and hot food)	Yes, no
	47	In general, how do you describe your eating habits as? (I like sweet and sour food)	Yes, no
	48	In general, how do you describe your eating habits as? (I like cold and refrigerated food)	Yes, no
	49	In general, how do you describe your eating habits as? (I tend to frequently snack)	Yes, no
	50	How would you describe your overnight sleep DURING this lock-down period?	Very good, good, OK, bad, very bad
	51	How would you describe your overnight sleep BEFORE this lock-down period?	Very good, good, OK, bad, very bad
	52	What activity were you engaged with BEFORE this lock-down period?	Did yoga, went fitness, went walking, did household, other
	53	What activity are you engaged with DURING this lock-down period?	Doing yoga, going fitness, going walk, doing household, other
	54	How much time do you spend for a structured physical activity as mentioned above during this lock-down period?	Never, <30 min, 30 min−1 h, >1 h
	55	Duration of practices per week during this lock-down period? (*Asana*)	Don't practice, <2 h, 2–4 h, 4–6 h, >6 h
	56	Duration of practices per week during this lock-down period? (*Pranayama*)	Don't practice, <2 h, 2–4 h, 4–6 h, >6 h
	57	Duration of practices per week during this lock-down period? (Meditation)	Don't practice, <2 h, 2–4 h, 4–6 h, >6 h
	58	Duration of practices per week during this lock-down period? (Religious practices)	Don't practice, <2 h, 2–4 h, 4–6 h, >6 h
	59	How motivated are/were you to start Yoga during this lock-down period?	Not at all, somewhat, very much so
	60	In general, how do you rate the happiness/peace you derive from the following? (Yoga and/ or religious practices)	Not at all, somewhat, very much
	61	In general, how do you rate the happiness/peace you derive from the following? (Money)	Not at all, somewhat, very much
	62	In general, how do you rate the happiness/peace you derive from the following? (Sensory pleasures)	Not at all, somewhat, very much
	63	In general, how do you rate the happiness/peace you derive from the following? (Name and fame)	Not at all, somewhat, very much
	64	In general, how do you rate the happiness/peace you derive from the following? (Service to society)	Not at all, somewhat, very much

Questions 11–15 accessed COVID-19 exposure of participants; these included self-reported symptoms, travel history, details of interaction with COVID-19–positive patient, and quarantine history. Physical health was accessed by rating physical strength and endurance (question 16) and disease history (question 17). In question 16, two extreme options were considered as a single option during analysis.

Twelve questions (questions 18–29) were included in CHAS to assess the mental health during the lockdown. The questions were designed to evaluate fear and anxiety during the lockdown and evaluate the individual's general personality or character. Standard neuropsychological questionnaires were not used to evaluate stress and anxiety.

The coping ability of participants was accessed by a direct question with four options, i.e., “Poor,” “Average,” “Very good,” and “Excellent” (question 30). During analysis, “Poor” and “Average” were merged into a single attribute, i.e., “Poor.” Similarly, “Very Good” and “Excellent” were merged to constitute “Good.” Questions 31–38 enquired about different activities of participants during lockdown; these questions indicate coping strategy of participants during lockdown. Questions 39–42 in lifestyle domain also provide information on coping strategy.

### Data Collection and Study Participants

The survey items for CHAS were prepared on Google forms and circulated in public through social media. Snowball method was used to acquire the data nationwide. Phone calls and special requests were sent to different sections of the society (~200 universities, Corporate companies, healthcare institutions, government organizations, wellness centers, and their networks) to acquire data within this short period. No inclusion and exclusion criteria were defined during the circulation of CHAS. Hence, the received responses showed diversity in age, gender, occupation, education, and other demographics (Table 2). This study was sponsored and conducted by S-VYASA. The participation was voluntary, and the response sheets were downloaded daily.

**Table 2:** Demographic characteristics and COVID-19 exposure in non-Yoga and Yoga groups.

**Demographics**	**Non-Yoga (*N* = 3,377), No. (%)**	**Yoga (*N* = 9,840), No. (%)**	**Total *N* = 13,217, No. (%)**	***P*-value**
**Gender**				<0.001
Female	1,097 (32.5)	4,397 (44.7)	5,494 (41.6)	
Male	2,280 (67.5)	5,443 (55.3)	7,723 (58.4)	
**Age**				<0.001
<20 years	372 (11.0)	332 (3.4)	704 (5.3)	
20–30 years	1,704 (50.5)	2,240 (22.8)	3,944 (29.8)	
31–40 years	761 (22.5)	2,375 (24.1)	3,136 (23.7)	
41–50 years	341 (10.1)	2,243 (22.8)	2,584 (19.6)	
51–60 years	136 (4.0)	1,519 (15.4)	1,655 (12.5)	
61–70 years	49 (1.5)	880 (8.9)	929 (7.0)	
71–80 years	12 (0.4)	209 (2.1)	221 (1.7)	
Above 80 years	2 (0.1)	42 (0.4)	44 (0.3)	
**Occupation**				<0.001
Agriculture	104 (3.1)	245 (2.5)	349 (2.6)	
Business	99 (2.9)	708 (7.2)	807 (6.1)	
Employed	1,209 (35.8)	2,809 (28.5)	4,018 (30.4)	
Homemaker	123 (3.6)	1,504 (15.3)	1,627 (12.3)	
Retired	56 (1.7)	744 (7.6)	800 (6.1)	
Student	1,248 (37.0)	1,358 (13.8)	2,606 (19.7)	
Professional	280 (8.3)	1,171 (11.9)	1,451 (11.0)	
Other	258 (7.6)	1,301 (13.2)	1,559 (11.8)	
**Education**				<0.001
Less than graduation	992 (29.4)	2,459 (25.0)	3,451 (26.1)	
Graduate	1,398 (41.4)	3,748 (38.1)	5,146 (38.9)	
Post graduate	987 (29.2)	3,633 (36.9)	4,620 (35.0)	
**Lockdown stay status**				<0.001
Family	2,799 (82.9)	8,429 (85.7)	11,228 (85.0)	
Friends	64 (1.9)	113 (1.1)	177 (1.3)	
Colleagues	193 (5.7)	332 (3.4)	525 (4.0)	
Alone	169 (5.0)	588 (6.0)	757 (5.7)	
Missing data	152 (4.5)	378 (3.8)	530 (4.0)	
**Working status during lockdown**				<0.001
Working from home	1,276 (37.8)	4,027 (40.9)	5,303 (40.1)	
Working from office	867 (25.7)	1,494 (15.2)	2,361 (17.9)	
Not working	1,234 (36.5)	4,319 (43.9)	5,553 (42.0)	
**COVID-19 symptoms**				<0.001
No symptoms	2,943 (87.1)	9,037 (91.8)	11,980 (90.7)	
Cough	68 (2.0)	78 (0.8)	146 (1.1)	
Fever	5 (0.1)	2 (0.0)	7 (0.0)	
Breathing difficulty	8 (0.2)	20 (0.2)	28 (0.2)	
Other	353 (10.5)	703 (7.1)	1,056 (8.0)	
**International travel since January 2020**				0.383
Yes	65 (1.9)	214 (2.2)	279 (2.1)	
No	3,312 (98.1)	9,626 (97.8)	12,938 (97.9)	
**Exposure to COVID-19**				<0.001
No exposure	3,106 (92.0)	9,381 (95.3)	12,487 (94.5)	
Recent COVID-19 interaction	19 (0.6)	40 (0.4)	59 (0.4)	
Were in quarantine	90 (2.7)	166 (1.7)	256 (1.9)	
Still in quarantine	54 (1.6)	99 (1.0)	153 (1.2)	
Healthcare worker	104 (3.1)	143 (1.5)	247 (1.9)	
Hospitalized	4 (0.1)	11 (0.1)	15 (0.1)	

The CHAS data were collected between May 9, 2020 and May 31, 2020, and 23,760 responses were received. Incomplete and unreliable responses, respondents from outside of India and respondents aged <18 years were excluded (*n* = 470). The remaining 23,290 responses were evaluated to assign participants in Yoga and non-Yoga groups. Inclusion criteria were age should be ≥18 years and all respondents should be residing in India. Yoga and non-Yoga group was defined according to the responses of question numbers 52, 53, 55, 56, and 57 of the CHAS questionnaire (Table 1).

#### Criteria for Defining Yoga Group

The Yoga group was defined as individuals who performed Yoga both before (question 52) and during (question 53) the lockdown, which included practicing one or more among *Asanas* (question 55) (Yoga postures), *Pranayama* (Yogic breathing exercises) (question 56), and meditation (question 57) for a few hours to more than 6 h per week during the lockdown. Participants, who replied “Did Yoga” for questions 52 and 53, but marked “Don't practice” for questions 55–57 were excluded from the Yoga group. According to these criteria, 9,840 participants qualified for the Yoga group.

Furthermore, the Yoga group was divided into four sub-groups, i.e., Yoga practitioners who practiced *Asana, Pranayama*, and meditation (all three together; *n* = 6,156), practitioners who practiced only *Asana* (*n* = 149), only *Pranayama* (*n* = 89), and only meditation (*n* = 1,485). The combination of two practices among *Asana, Pranayama*, and meditation was not considered as a sub-group.

#### Criteria for Defining Non-Yoga Group

The non-Yoga group included respondents who did not perform Yoga before (question 52) or during (question 53) the lockdown and replied “Don't Practice” for the questions on *Asana* (question 55), *Pranayama* (question 56), and meditation (question 57). Following the aforementioned inclusion criteria, 3,377 participants were accepted in the non-Yoga group.

### Statistical Analysis

R Statistical software, version 4.0.0, was used for data cleaning, extraction, and analyses. The arsenal package in R was used to determine cross-tabulations and χ^2^ test; logistic and multinomial regression was used. Age, gender, occupation, education, and working status during lockdown were used as covariates.

The dependent variables were the study groups. We used multiple predictors in each of the regression models. Sequential contrast was used for ordinal variables. The predictors were selected based on the domains presented in the survey. The domains were demographic details, physical health, mental health, coping strategy, and lifestyle.

## Results

### Demographic Characterization

Table 2 summarizes the demographics of the non-Yoga group (25.6%), Yoga group (74.4%), and total participants. Participation of males in the survey was proportionally higher in both non-Yoga (67.5%) and Yoga (55.3%) groups. The young population in the age group of 20–30 years was higher in the non-Yoga (50.5%) group than in the Yoga group (22.8%). The participation from the age group > 50 years was higher in the Yoga group (26.8%) than in the non-Yoga group (6.0%). Our data also revealed that the percentage of employed and professional participants was higher in both groups, with 44.1% in non-Yoga and 40.4% in Yoga. The non-Yoga group had 37.0% participation from young students. Most of the participants had a good educational background as they were either graduates or post-graduates. We noted that 85.0% of the participants stayed with their family during the lockdown, apparently lending help to cope with stress. On further analysis, we found that the non-working participants were fewer in the non-Yoga group (36.5 vs. 43.9%). Furthermore, the proportion of participants going to the office during lockdown was more in the non-Yoga group (25.7 vs. 15.2%).

### COVID-19 Exposure

Participants with no symptoms are less likely to be in the non-Yoga group than participants with cough, fever, breathing difficulty, and other symptoms (Table 2). The symptoms of COVID-19 were self-reported. Approximately 98.1% of non-Yoga and 97.8% of the Yoga group did not undertake any international travel since January 2020. Further, the non-Yoga group is more likely to have exposure to COVID-19 than the Yoga group.

### Lifestyle

Although the proportion of participants using substances was lower in both groups, the non-Yoga group was more likely to depend on alcohol, tobacco, and other substances (Table 3). The non-Yoga group was less likely to have a good quality of sleep before and during the lockdown than the Yoga group, odds ratio (OR) <1 (Table 3).

**Table 3:** Lifestyle in non-Yoga and Yoga groups.

**Lifestyle**			**Non-Yoga (*N* = 3,377) Reference**	**Yoga (*N* = 9,840)**
**Substance abuse** No. (%) Adjusted *p*-value Adjusted OR (95% CI) Unadjusted OR (95% CI)	Tobacco	Never	2,961 (87.7)	9,586 (97.4)
		Occasionally	288 (8.5)	191 (1.9) 0.001 0.58 (0.42–0.80) 0.64 (0.47–0.87)
		Regularly	128 (3.8)	63 (0.6) 0.067 0.57 (0.31–1.03) 0.59 (0.32–1.05)
	Alcohol	Never	2,703 (80.0)	9,499 (96.5)
		Occasionally	636 (18.8)	317 (3.2) <0.001 0.56 (0.44–0.72) 0.59 (0.46–0.75)
		Regularly	38 (1.1)	24 (0.2) 0.010 3.08 (1.29–7.14) 3.33 (1.38–7.80)
	Other substances	Never	3,261 (96.6)	9,688 (98.5)
		Occasionally	74 (2.2)	113 (1.1) 0.090 1.53 (0.94–2.51) 1.41 (0.87–2.30)
		Regularly	42 (1.2)	39 (0.4) 0.229 0.58 (0.24–1.39) 0.51 (0.21–1.23)
	Increased substance dependence	Yes	63 (1.9)	55 (0.6)
		No	1,229 (36.4)	1,917 (19.5) <0.001 0.32 (0.18–0.58) 0.30 (0.17–0.55)
		Not applicable	2,085 (61.7)	7,868 (80.0) <0.001 0.27 (0.15–0.48) 0.26 (0.14–0.47)
**Eating habits** No. (%) Adjusted *p*-value Adjusted OR (95% CI) Unadjusted OR (95% CI)	Disciplined to time	Yes	2,414 (71.5)	8,915 (90.6)
		No	963 (28.5)	925 (9.4) <0.001 0.60 (0.51–0.52) 0.58 (0.49–0.69)
	Vegetarian/vegan	Yes	1,373 (40.7)	8,713 (88.5)
		No	2,004 (59.3)	1,127 (11.5) <0.001 0.31 (0.27–0.36) 0.27 (0.23–0.31)
	Junk food	Yes	1,505 (44.6)	1,166 (11.8)
		No	1,872 (55.4)	8,674 (88.2) <0.001 1.39 (1.18–1.64) 1.69 (1.44–1.98)
	Spicy and hot food	Yes	2,322 (68.8)	2,992 (30.4)
		No	1,055 (31.2)	6,848 (69.6) <0.001 1.77 (1.53–2.05) 1.91 (1.65–2.21)
	Sweet and sour food	Yes	2,225 (65.9)	5,234 (53.2)
		No	1,152 (34.1)	4,606 (46.8) 0.115 1.12 (0.97–1.30) 1.15 (1.00–1.33)
	Cold and refrigerated food	Yes	1,123 (33.3)	1,019 (10.4)
		No	2,254 (66.7)	8,821 (89.6) 0.993 (0.84–1.19) 1.09 (0.92–1.30)
	Frequent snacks	Yes	1,758 (52.1)	2,729 (27.7)
		No	1,619 (47.9)	7,111 (72.3) 0.235 0.92 (0.79–1.06) 0.98 (0.85–1.12)
**Sleep** No. (%) Adjusted *p*-value Adjusted OR (95% CI) Unadjusted OR (95% CI)	During lockdown period	Good	2,171 (64.3)	8,558 (87.0)
		Ok	856 (25.3)	1,107 (11.2) <0.001 0.67 (0.55–0.81) 0.67 (0.55–0.81)
		Bad	350 (10.4)	175 (1.8) 0.011 0.64 (0.45–0.9) 0.60 (0.43–0.85)
	Before lockdown period	Good	2,427 (71.9)	8,744 (88.9)
		Ok	776 (23.0)	992 (10.1) 0.035 0.81 (0.66–0.99) 0.81 (0.67–0.98)
		**Bad**	174 (5.2)	104 (1.1) 0.387 0.81 (0.51–1.29) 0.76 (0.48–1.21)
**Happiness/peace** No. (%) Adjusted *p*-value Adjusted OR (95% CI) Unadjusted OR (95% CI)	By doing Yoga/religious practices	Not at all	1,358 (40.2)	184 (1.9)
		Somewhat	1,391 (41.2)	1,795 (18.2) <0.001 5.88 (4.63–7.52) 6.05 (4.78–7.70)
		Very much	628 (18.6)	7,861 (79.9) <0.001 2.42 (2.07–2.82) 2.54 (2.18–2.95)
	By earning money	Not at all	688 (20.4)	3,344 (34.0)
		Somewhat	1,760 (52.1)	5,456 (55.4) <0.001 0.68 (0.57–0.82) 0.70 (0.59–0.84)
		Very much	929 (27.5)	1,040 (10.6) <0.001 0.56 (0.46–0.68) 0.54 (0.45–0.66)
	By sensory pleasure	Not at all	764 (22.6)	3,727 (37.9)
		Somewhat	1,895 (56.1)	4,330 (44.0) <0.001 0.71 (0.59–0.85) 0.72 (0.60–0.86)
		Very much	718 (21.3)	1,783 (18.1) 0.698 0.96 (0.79–1.17) 0.98 (0.81–1.19)
	By name and fame	Not at all	1,084 (32.1)	5,705 (58.0)
		Somewhat	1,540 (45.6)	3,033 (30.8) 0.020 0.81 (0.68–0.97) 0.73 (0.62–0.87)
		Very much	753 (22.3)	1,102 (11.2) 0.840 0.98 (0.79–1.21) 0.96 (0.78–1.19)
	By social service	Not at all	361 (10.7)	401 (4.1)
		Somewhat	1,460 (43.2)	3,157 (32.1) 0.292 1.17 (0.88–1.55) 1.22 (0.93–1.61)
		Very much	1,556 (46.1)	6,282 (63.8) 0.970 (0.86–1.16) 0.92 (0.80–1.07)
**Motivation to start/practice more Yoga during lockdown** No. (%) Adjusted *p*-value Adjusted OR (95% CI) Unadjusted OR (95% CI)		Not at all	1,508 (44.7)	395 (4.0)
		Somewhat	1,665 (49.3)	2,405 (24.4) <0.001 4.54 (3.79–5.46) 4.22 (3.54–5.03)
		Very much	204 (6.0)	7,040 (71.5) <0.001 11.39 (9.50–13.72) 11.33 (9.47–13.62)
**Any religious practices other than Yoga** No. (%) Adjusted *p*-value Adjusted OR (95% CI) Unadjusted OR (95% CI)		Never	2,002 (59.3)	3,155 (32.1)
		<4 h	1,331 (39.4)	5,658 (57.5) <0.001 1.79 (1.56–2.06) 1.82 (1.59–2.09)
		>4 h	44 (1.3)	1,027 (10.4) <0.001 3.09 (2.04–4.80) 3.13 (2.08–4.80)

*Sequential contrast was used for ordinal variables and reference level is mentioned in the table for nominal variables*.

Participants of the non-Yoga group were less likely to have food in a disciplined manner [unadjusted OR = 0.58 (0.49–0.69), adjusted OR = 0.60 (0.51–0.52)] and were less likely to be vegetarian [unadjusted OR = 0.27 (0.23–0.31), adjusted OR = 0.31 (0.27–0.36)] (Table 3). The non-Yoga group was more likely to consume junk food [unadjusted OR = 1.69 (1.44–1.98), adjusted OR = 1.39 (1.18–1.64)] and spicy and hot food [unadjusted OR = 1.91 (1.65–2.21), adjusted OR = 1.77 (1.53–2.05)]. Interestingly, about 55.3% of the non-Yoga participants were motivated to start Yoga during the lockdown (Table 3).

### Physical Health

The Yoga group reported very good physical strength and endurance, with only 7.5% of participants reporting an average or below average physical strength and endurance (Table 4). The non-Yoga practitioners were less likely to have good physical strength and endurance (OR < 1), suggesting physical endurance attributes might be superior in Yoga practitioners. Disease risk for co-morbidities, including heart diseases, lung disease, blood pressure, and others, was lower in both the groups (Table 4).

**Table 4:** Physical health in non-Yoga and Yoga groups.

**Physical health parameters**	**Non-Yoga (*N* = 3,377), No. (%) (Reference)**	**Yoga (*N* = 9,840), No. (%)**	**Non-Yoga vs. Yoga, unadjusted odds ratio (95% CI)**	**Non-Yoga vs. Yoga, adjusted odds ratio (95% CI)**	**Adjusted *p*-value**
**Physical strength and endurance**					
Good	2,688 (79.6)	9,106 (92.5)			
Average	630 (18.7)	697 (7.1)	0.31 (0.28–0.35)	0.32 (0.28–0.37)	<0.001
Bad	59 (1.7)	37 (0.4)	0.51 (0.32–0.79)	0.59 (0.35–0.98)	0.045
**Disease history**					
High risk	391 (11.6)	1,739 (17.7)	0.53 (0.46–0.60)	1.08 (0.94)	0.286
No risk	2,986 (88.4)	8,101 (82.3)			

*Sequential contrast was used for ordinal variables and reference level is mentioned in the table for nominal variables*.

### Mental Health

**Figure 1:**
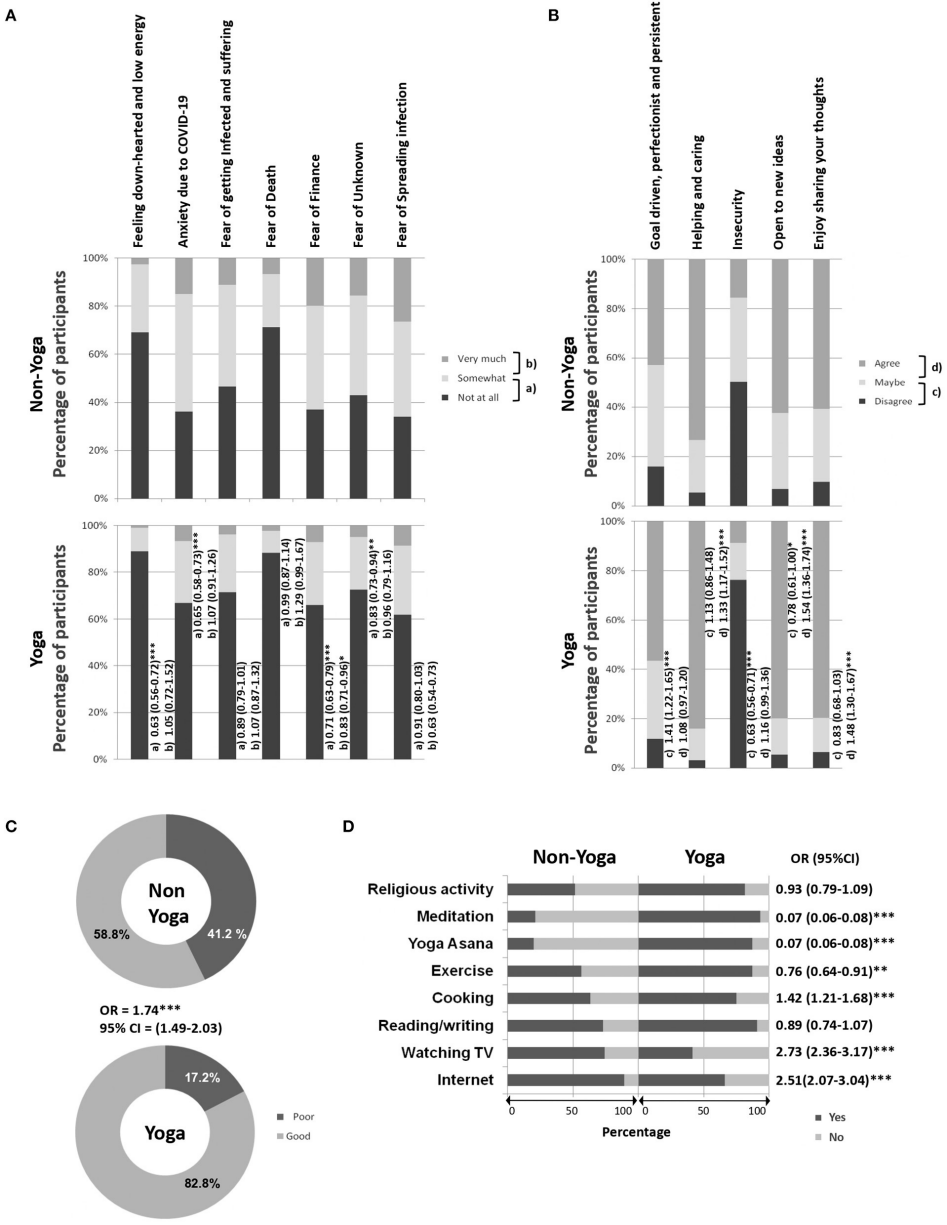
Mental health and coping strategy of non-Yoga and Yoga groups. **(A)** Anxiety and fear during lockdown, **(B)** general personality, **(C)** coping ability, and **(D)** coping activities. In **(A,B)**, odds ratio and CI (in brackets) for each parameter are mentioned on the right side of the bar. The small alphabets represent the pair of tested variables when Yoga group is compared with non-Yoga group. The representation are (a) not at all vs. somewhat, (b) somewhat vs. very much, (c) disagree vs. maybe, and (d) maybe vs. agree. Sequential contrast was used for ordinal variables. *p < 0.05, **p < 0.01, ***p < 0.001.

The non-Yoga group reported higher anxiety and fear than the Yoga group when asked about “down-hearted” feeling, low energy (30.9%), anxiety due to COVID-19 (63.9%), fear of getting infected, related suffering (53.4%), fear of death (28.7%), fear of financial difficulties (63.0%), fear of the unknown (57.0%), and fear of spreading infection (65.9%) (Figure 1A).

Furthermore, the non-Yoga group was less goal-driven and oriented toward perfection in their activities (15.9%), less helpful and caring (5.2%), more insecure (50.2%), not open to new ideas (6.6%), and do not enjoy sharing their thoughts (9.7%) than the Yoga group (Figure 1B).

### Strategies for Coping With COVID-19 and Lockdown-Related Stress

Most of the Yoga group members reported good coping ability (82.8%), while most of the non-Yoga (58.8%) group reported poor coping ability thereby highlighting a significant difference in two groups (Figure 1C).

Figure 1D shows that the non-Yoga group could cope using the Internet, watching TV, reading/writing, cooking, religious activity, and exercise (>50%). In contrast, the Yoga group was engaged in yoga *Asanas*, meditation, and religious/spiritual activities besides using the Internet, reading/writing, cooking, and exercise (>50%).

### Meditation Is Highly Effective to Bring Mental Stability and Strength

We also examined whether practicing a combination of *Asanas, Pranayama*, and meditation has a different influence on outcome variables than practicing one of these three yogic practices, individually. Table 5 summarizes the different parameters of physical health, mental health, and coping strategies of four sub-groups. Meditation was frequently performed by the age group of 41–50 years (26.3%), while *Asana* (49.7%) and *Pranayama* (39.3%) were favored by young people between the ages of 20 and 30 years. The consolidated Yoga practice was preferred mainly by participants of 31–40 years (24.5%).

**Table 5:** Physical health, mental health, and coping strategy among the different Yoga groups practicing all or a particular form of Yoga.

		**Combined Yoga group (*N* = 6,156)(Reference)**	**Only *Asana* (*N* = 149)**	**Only meditation (*N* = 1,485)**	**Only *Pranayama* (*N* = 89)**
Physical health No. (%) Adjusted *p*-value Adjusted OR (95% CI) Unadjusted OR (95% CI)	**Physical strength and endurance**				
	Good	5,729 (93.1)	124 (83.2)	1,388 (93.5)	85 (95.5)
	Average	405 (6.6)	23 (15.4)	89 (6.0)	4 (4.5)
			0.133	0.581	0.332
			2.28 (0.78–6.64)	0.85 (0.47–1.53)	0.76 (0.44–1.32)
			2.79 (0.96–8.07)	0.90 (0.50–1.62)	0.73 (0.00–2.34E06)
	Bad	22 (0.4)	2 (1.3)	8 (0.5)	0 (0.0)
			0.074	0.009	<0.001
			1.92 (0.94–3.91)	1.69 (1.14–2.49)	0.00 (0.00–0.00)
			1.91 (0.94–3.88)	2.03 (1.37–2.99)	0.05 (0.00–271.11)
	**Disease history**				
	High risk	1,076 (17.5)	20 (13.4)	287 (19.3)	15 (16.9)
	No risk	5,080 (82.5)	129 (86.6)	1,198 (80.7)	74 (83.1)
			0.588	0.892	0.287
			0.87 (0.52–1.45)	0.99 (0.84–1.16)	0.72 (0.40–1.31)
			1.71 (1.02–2.85)	0.86 (0.74–1.00)	1.10 (0.60–2.01)
Mental health No. (%) Adjusted *p*-value Adjusted OR (95% CI) Unadjusted OR (95% CI)	**Down-hearted and low energy**				
	Not at all	5,485 (89.1)	109 (73.2)	1,385 (93.3)	72 (80.9)
	Somewhat	620 (10.1)	36 (24.2)	91 (6.1)	16 (18.0)
			0.580	0.803	0.500
			1.25 (0.56–2.78)	1.07 (0.64–1.78)	1.76 (0.34–9.09)
			1.31 (0.59–2.91)	1.13 (0.67–1.90)	1.33 (0.31–5.69)
	Very much	51 (0.8)	4 (2.7)	9 (0.6)	1 (1.1)
			0.926	0.189	0.526
			1.03 (0.60–1.77)	1.27 (0.89–1.81)	0.71 (0.25–2.04)
			1.26 (0.73–2.16)	0.90 (0.63–1.29)	0.53 (0.21–1.35)
	**Anxiety due to COVID-19**				
	Not at all	4,001 (65.0)	62 (41.6)	1,197 (80.6)	45 (50.6)
	Somewhat	1,752 (28.5)	65 (43.6)	230 (15.5)	27 (30.3)
			0.430	0.003	0.347
			1.19 (0.77–1.84)	0.71 (0.57–0.89)	0.78 (0.47–1.31)
			1.26 (0.82–1.95)	0.70 (0.57–0.88)	0.84 (0.51–1.38)
	Very much	403 (6.5)	22 (14.8)	58 (3.9)	17 (19.1)
			0.218	0.081	<0.001
			1.24 (0.88–1.73)	1.17 (0.98–1.40)	2.48 (1.57–3.92)
			1.36 (0.98–1.89)	1.15 (0.97–1.37)	2.79 (1.80–4.33)
	**Fear of getting infected**				
	Not at all	4,260 (69.2)	67 (45.0)	1,254 (84.4)	51 (57.3)
	Somewhat	1,649 (26.8)	67 (45.0)	210 (14.1)	33 (37.1)
			0.153	0.423	0.415
			1.54 (0.85–2.77)	0.85 (0.56–1.27)	0.68 (0.27–1.73)
			1.57 (0.87–2.82)	0.80 (0.54–1.20)	0.79 (0.33–1.90)
	Very much	247 (4.0)	15 (10.1)	21 (1.4)	5 (5.6)
			0.453	0.566	<0.001
			1.15 (0.79–1.68)	0.93 (0.72–1.20)	0.20 (0.12–0.36)
			1.03 (0.71–1.50)	0.96 (0.74–1.23)	0.27 (0.16–0.47)
	**Fear of death**				
	Not at all	5,393 (87.6)	120 (80.5)	1,411 (95.0)	61 (68.5)
	Somewhat	614 (10.0)	23 (15.4)	62 (4.2)	18 (20.2)
			0.143	0.546	0.282
			0.58 (0.29–1.20)	0.85 (0.50–1.44)	1.48 (0.72–3.03)
			0.56 (0.28–1.15)	0.86 (0.52–1.43)	1.98 (0.98–4.01)
	Very much	149 (2.4)	6 (4.0)	12 (0.8)	10 (11.2)
			0.061	0.833	<0.001
			0.61 (0.37–1.02)	1.04 (0.73–1.49)	4.38 (2.45–7.81)
			0.62 (0.37–1.03)	1.13 (0.80–1.61)	3.17 (1.85–5.46)
	**Fear of finance**				
	Not at all	3,931 (63.9)	59 (39.6)	1,182 (79.6)	46 (51.7)
	Somewhat	1,777 (28.9)	62 (41.6)	261 (17.6)	32 (36.0)
			0.068	0.043	0.952
			1.49 (0.97–2.28)	0.76 (0.58–0.99)	1.02 (0.53–1.95)
			1.48 (0.98–2.24)	0.74 (0.57–0.96)	1.00 (0.54–1.87)
	Very much	448 (7.3)	28 (18.8)	42 (2.8)	11 (12.4)
			0.226	0.007	0.117
			1.21 (0.89–1.65)	0.78 (0.65–0.93)	0.69 (0.44–1.10)
			1.26 (0.93–1.71)	0.81 (0.68–0.97)	0.80 (0.51–1.24)
	**Fear of unknown**				
	Not at all	4,366 (70.9)	69 (46.3)	1,275 (85.9)	44 (49.4)
	Somewhat	1,486 (24.1)	57 (38.3)	184 (12.4)	34 (38.2)
			0.532	0.326	0.169
			1.19 (0.70–2.02)	0.83 (0.56–1.21)	1.70 (0.80–3.63)
			1.41 (0.83–2.40)	0.80 (0.55–1.17)	2.00 (0.95–4.23)
	Very much	304 (4.9)	23 (15.4)	26 (1.8)	11 (12.4)
			0.015	0.764	0.121
			1.56 (1.09–2.23)	0.96 (0.75–1.23)	1.48 (0.90–2.41)
			1.63 (1.14–2.31)	1.07 (0.84–1.36)	1.30 (0.80–2.10)
	**Fear of spread**				
	Not at all	3,665 (59.5)	65 (43.6)	1,127 (75.9)	42 (47.2)
	Somewhat	1,933 (31.4)	56 (37.6)	309 (20.8)	32 (36.0)
			0.634	0.300	0.908
			0.89 (0.56–1.42)	0.87 (0.66–1.14)	1.04 (0.54–2.01)
			0.75 (0.47–1.20)	0.83 (0.63–1.08)	0.99 (0.52–1.87)
	Very much	558 (9.1)	28 (18.8)	49 (3.3)	15 (16.9)
			0.545	0.010	0.347
			0.90 (0.65–1.26)	0.79 (0.66–0.94)	0.80 (0.51–1.27)
			1.02 (0.73–1.42)	0.75 (0.63–0.90)	0.80 (0.51–1.24)
	**Goal driven, perfectionist, and persistent**				
	Disagree	715 (11.6)	20 (13.4)	181 (12.2)	15 (16.9)
	Maybe	1,963 (31.9)	59 (39.6)	422 (28.4)	31 (34.8)
			0.541	0.260	0.738
			0.88 (0.59–1.31)	1.08 (0.94–1.25)	0.91 (0.54–1.55)
			0.80 (0.55–1.19)	1.04 (0.91–1.20)	0.73 (0.45–1.17)
	Agree	3,478 (56.5)	70 (47.0)	882 (59.4)	43 (48.3)
			0.387	0.124	0.572
			0.87 (0.63–1.19)	1.10 (0.97–1.24)	1.13 (0.74–1.73)
			0.88 (0.64–1.21)	1.03 (0.91–1.16)	0.92 (0.61–1.38)
	**Helping and caring**				
	Disagree	199 (3.2)	6 (4.0)	41 (2.8)	6 (6.7)
	Maybe	751 (12.2)	24 (16.1)	169 (11.4)	23 (25.8)
			0.840	0.053	0.760
			0.93 (0.46–1.87)	1.32 (1.00–1.75)	1.12 (0.53–2.38)
			0.96 (0.49–1.88)	1.55 (1.17–2.05)	1.18 (0.56–2.51)
	Agree	5,206 (84.6)	119 (79.9)	1,275 (85.9)	60 (67.4)
			0.481	0.863	<0.001
			1.22 (0.71–2.10)	0.98 (0.79–1.22)	0.32 (0.18–0.56)
			1.10 (0.66–1.84)	0.93 (0.75–1.15)	0.38 (0.22–0.66)
	**Insecurity**				
	Disagree	4,651 (75.6)	73 (49.0)	1,261 (84.9)	59 (66.3)
	Maybe	977 (15.9)	50 (33.6)	114 (7.7)	21 (23.6)
			0.001	<0.001	0.326
			1.85 (1.29–2.67)	0.60 (0.51–0.70)	1.30 (0.77–2.22)
			2.06 (1.45–2.94)	0.61 (0.52–0.72)	1.17 (0.69–1.99)
	Agree	528 (8.6)	26 (17.4)	110 (7.4)	9 (10.1)
			0.886	<0.001	0.184
			1.02 (0.74–1.41)	1.52 (1.25–1.84)	0.71 (0.43–1.18)
			1.04 (0.76–1.43)	1.53 (1.27–1.85)	0.82 (0.50–1.34)
	**Open to new ideas**				
	Disagree	319 (5.2)	8 (5.4)	91 (6.1)	6 (6.7)
	Maybe	893 (14.5)	38 (25.5)	196 (13.2)	13 (14.6)
			0.067	0.879	0.081
			1.87 (0.96–3.66)	0.98 (0.78–1.24)	2.05 (0.92–4.59)
			2.07 (1.09–3.91)	0.90 (0.72–1.13)	1.86 (0.84–4.13)
	Agree	4,944 (80.3)	103 (69.1)	1,198 (80.7)	70 (78.7)
			0.237	0.183	0.147
			0.75 (0.47–1.21)	0.88 (0.73–1.06)	1.63 (0.84–3.17)
			0.75 (0.47–1.18)	0.88 (0.73–1.06)	1.90 (0.99–3.65)
	**Enjoy sharing your thoughts**				
	Disagree	361 (5.9)	17 (11.4)	96 (6.5)	15 (16.9)
	Maybe	854 (13.9)	33 (22.1)	164 (11.0)	12 (13.5)
			0.002	0.158	<0.001
			0.48 (0.30–0.76)	0.86 (0.69–1.06)	0.21 (0.12–0.35)
			0.54 (0.35–0.85)	0.86 (0.70–1.07)	0.20 (0.12–0.34)
	Agree	4,941 (80.3)	99 (66.4)	1,225 (82.5)	62 (69.7)
			0.386	0.632	0.253
			0.83 (0.55–1.26)	1.05 (0.86–1.27)	1.45 (0.77–2.74)
			0.71 (0.48–1.07)	1.12 (0.93–1.35)	1.23 (0.67–2.24)
Coping strategy No. (%) Adjusted *p*-value Adjusted OR (95% CI) Unadjusted OR (95% CI)	**Coping ability**				
	Poor	1,037 (16.8)	50 (33.6)	219 (14.7)	18 (20.2)
	Good	5,119 (83.2)	99 (66.4)	1,266 (85.3)	71 (79.8)
			0.163	0.006	0.472
			0.80 (0.59–1.10)	1.23 (1.06–1.42)	0.86 (0.58–1.29)
			0.64 (0.48–0.86)	1.23 (1.07–1.42)	1.09 (0.74–1.61)
	**Do you prefer watching TV**				
	Yes	2,698 (43.8)	93 (62.4)	449 (30.2)	59 (66.3)
	No	3,458 (56.2)	56 (37.6)	1,036 (69.8)	30 (33.7)
			0.037	<0.001	0.001
			0.64 (0.42–0.97)	1.38 (1.18–1.62)	0.43 (0.26–0.71)
			0.68 (0.46–1.01)	1.38 (1.18–1.61)	0.41 (0.26–0.67)
	**Do you prefer reading/writing**				
	Yes	5,677 (92.2)	116 (77.9)	1,337 (90.0)	71 (79.8)
	No	479 (7.8)	33 (22.1)	148 (10.0)	18 (20.2)
			0.051	<0.001	0.959
			1.72 (1.00–2.95)	0.52 (0.39–0.70)	1.02 (0.52–2.01)
			1.63 72 (1.00–2.95)	0.57 (0.43–0.76)	1.24 (0.66–2.30)
	**Do you prefer cooking**				
	Yes	4,662 (75.7)	110 (73.8)	1,094 (73.7)	57 (64.0)
	No	1,494 (24.3)	39 (26.2)	391 (26.3)	32 (36.0)
			0.496	0.061	0.988
			0.84 (0.52–1.38)	0.84 (0.69–1.01)	1.00 (0.59–1.68)
			0.73 (0.47–1.13)	0.80 (0.68–0.95)	1.15 (0.71–1.86)
	**Do you prefer exercise**				
	Yes	5,739 (93.2)	134 (89.9)	919 (61.9)	74 (83.1)
	No	417 (6.8)	15 (10.1)	566 (38.1)	15 (16.9)
			0.008	<0.001	0.942
			0.40 (0.21–0.79)	3.96 (3.27–4.79)	1.03 (0.51–2.07)
			0.41 (0.22–0.79)	4.18 (3.46–5.04)	0.92 (0.47–1.80)
	**Do you prefer** ***Yoga Asana***				
	Yes	5,965 (96.9)	135 (90.6)	744 (50.1)	74 (83.1)
	No	191 (3.1)	14 (9.4)	741 (49.9)	15 (16.9)
			0.242	<0.001	0.121
			0.64 (0.31–1.35)	24.31 (19.85–29.78)	1.84 (0.85–3.98)
			0.79 (0.39–1.61)	24.78 (20.29–30.27)	1.92 (0.93–3.95)
	**Do you prefer meditation**				
	Yes	5,984 (97.2)	50 (33.6)	1,433 (96.5)	51 (57.3)
	No	172 (2.8)	99 (66.4)	52 (3.5)	38 (42.7)
			<0.001	<0.001	<0.001
			58.60 (37.77–90.90)	0.23 (0.14–0.36)	19.01 (11.05–32.70)
			62.43 (41.25–94.49)	0.21 (0.13–0.32)	19.15 (11.42–32.11)
	**Do you prefer religious activity**				
	Yes	5,305 (86.2)	89 (59.7)	1,121 (75.5)	62 (69.7)
	No	851 (13.8)	60 (40.3)	364 (24.5)	27 (30.3)
			0.026	0.009	0.057
			1.66 (1.06–2.59)	1.28 (1.06–1.54)	1.69 (0.99–2.91)
			1.74 (1.14–2.65)	1.25 (1.04–1.51)	1.39 (0.83–2.33)
	**Do you prefer Internet**				
	Yes	4,247 (69.0)	131 (87.9)	806 (54.3)	68 (76.4)
	No	1,909 (31.0)	18 (12.1)	679 (45.7)	21 (23.6)
			0.039	0.022	0.994
			0.54 (0.31–0.97)	1.20 (1.03–1.41)	1.00 (0.56–1.78)
			0.41 (0.24–0.71)	1.34 (1.15–1.56)	0.84 (0.49–1.45)

*Sequential contrast was used for ordinal variables and reference level is mentioned in the table for nominal variables*.

Physical health, predicted by strength and endurance, revealed that the *Asana* group might have lower physical health, whereas co-morbidities were higher in the meditation group (19.3%). Good mental ability was revealed by lower anxiety and stress in the meditation group, followed by the combined group, *Asana* and *Pranayama*, sequentially. In addition, the ability to cope with the stress of COVID-19 was highest and comparable in the meditation group (85.3%) and the combined yoga group (83.2%). The preference of watching TV during lockdown was least in the meditation group (30.2%); their preferences included reading/writing (90.0%) and meditation (96.5%). The combined group preferred a range of activities, including reading/writing (92.2%), cooking (75.7%), exercise (93.2%), *Yogasana* (96.9%), meditation (97.1%), and religious activity (86.2%). TV watching was preferred by the *Pranayama* group (66.3%) followed by the *Asana* group (62.4%), and the Internet was preferred by the *Asana* group (87.9%) followed by the *Pranayama* group (76.4%). The aforementioned observations suggest that meditation might be more effective in reducing stress and anxiety and improving coping abilities in lockdown situations.

## Discussion

Despite limited public health intervention strategies, Yoga has remained the mainstay for improving well-being, disease risk reduction, and improving mental and physical health (17–20, 24, 25). We had earlier reported significant barriers in access to Yoga resources even though the prevalence of Yoga in India and elsewhere was significantly noteworthy (26, 27). Regardless, several studies have reported over time better physical health, mental health, and quality of life both among healthy individuals and those with disease or disorder (17–20, 24, 25). Yoga has been known to improve physical and mental health compared with a physically active group or a physically inactive group, yet the reliance on its anticipated benefits has never been assessed in any nationwide study during a health crisis (28, 29).

Yoga represents a regulated lifestyle that involves *Asanas, Pranayamas*, and meditation. It makes an individual self-aware of his/her body, mind, thoughts, and soul. The Yogic teaching is based on the fundamentals of *Yama* (restraints) and *Niyama* (observances) (30). *Yama* includes teachings of non-violence, truthfulness, non-stealing, moderation, and non-hoarding, whereas *Niyama* includes teachings of cleanliness, contentment, self-discipline, self-study, and wellness. Yoga practitioners routinely isolate themselves from the general population to achieve higher spiritual goals (31). Thus, one who follows the Yoga and Yogic lifestyle can easily maintain cleanliness and social distancing without an agitated mind. Therefore, Yoga practitioners can be hypothesized to quickly adapt to lockdown rules without experiencing chronic anxiety and stress.

The present study extends the above hypothesis operationalized as an investigation carried out when the world is gripped with fear and uncertainty due to an impending pandemic. In this context, the present study has evaluated the outcomes of physical and mental health, including lifestyle and coping strategies of Yoga and non-Yoga groups, during the lockdown imposed by COVID-19 pandemic. A CHAS questionnaire was generated following the Delphi protocol and was circulated among the Indian mass by snowball method as Google Forms. Phone calls and special requests were sent to different sections of the society including ~200 universities, Corporate companies, healthcare institutions, government organizations, wellness centers, and their networks to acquire data. The data were collected between May 9, 2020 and May 31, 2020; data were downloaded daily.

In the present survey, among the total respondents of 23,290, 42.2% (*n* = 9,840) were Yoga practitioners, which is much higher than the previous report of 11.8% of a nationwide randomized structured survey in 2017 (27). This may not indicate a true rise in the number of yoga practitioners in the country as this was not a randomly selected population.

The current study reports proportionately higher response from males than females. Non-Yoga group has a higher percentage of young participants than the Yoga group, which is a limitation of the study. In both Yoga and non-Yoga groups, most of the participants were employed or well-educated professionals. Furthermore, the analysis of the data collected during this survey revealed that both groups represented a similar proportion of participants living with family; however, a small proportion of participants were also reported to be living alone in both groups. Thus, loneliness cannot be a leading attribute of anxiety, stress, and fear in this study. Another feature of the participants who responded to the survey was that a greater proportion of the working and office going population was represented in the non-Yoga. Working and attending office during the lockdown may be considered as a reason for not practicing Yoga.

Emerging data have shown differences in the susceptibility to COVID-19 symptoms based on age and co-morbidities (4). Studies have shown the benefits of Yoga intervention in reducing the risk and severity of diabetes and other co-morbidities (24, 25). Such interventions may, therefore, be helpful for the risk reduction of COVID-19. Our data show increased non-Yoga group susceptibility to COVID-19 compared with those belonging to the Yoga group; however, RT-PCR for COVID-19 was not carried out to confirm this. Regardless, this calls for new public health and cost-effective intervention strategies based on a digital Yoga interface, compliant to Tele-Yoga regulations. Recently, a breathing technique known as Liuzijue has been reported to improve pulmonary function and quality of life in discharged COVID-19 patients (32). However, it cannot be neglected that the Yoga group is mainly constituted by participants who are “not working” or “working from home,” because of which they have a lower COVID-19 risk and are less fearful about COVID-19.

Sleeping and eating habits in the Yoga group were more health promotive. Furthermore, it also revealed that the dependence on substances use, as resources to cope with anxiety and stress, was lower in the Yoga group. Moreover, good physical strength and endurance coupled with the absence of chronic disease were also proportionately higher in the Yoga group, suggesting better health outcomes corresponding to COVID-19. Our study, however, did not estimate the non-Yoga exercise groups consisting of sportsmen or other physical activities.

We observed that the non-Yoga group was coping with COVID-19 lockdown by relying on the Internet, TV, reading/writing, cooking, and exercise, while the Yoga group was more engaged in *Asana*, meditation, and religious/spiritual activities besides using the Internet, reading/writing, cooking, and exercise. The Yoga group maintained their routine consistently, while the non-Yoga group could not sustain their regular practices.

The comparisons between the two groups unanimously showed that the non-Yoga group faced mental challenges. They reported higher anxiety and fear associated with COVID-19, including fear of getting infected with COVID-19, death, finance-related stress, and spreading COVID-19 in addition to other unknown causes. Instead, compared with the non-Yoga group, the Yoga group was reportedly more goal-driven and methodical, eliciting responses that showed a helping and caring attitude. The latter group displayed more openness to new ideas and enjoyed sharing their thoughts, being less insecure. Reports from China also show similar psychological distress (8, 17, 33).

COVID-19–related stress creates panic and reduces quality of life in patients as well as in healthcare workers (HCWs). Hence, introducing Yoga in the healthcare system would be beneficial for the patients, HCWs, and other service providers: they may successfully cope with psychological stress (34–37). Stress, anxiety, and depression among HCWs in COVID-19 pandemic was reduced by practicing *Sudarshan kriya* (38). Perceived stress in COVID-19 patients was also reduced by multimedia psychoeducational intervention, which included relaxation and mindfulness techniques (39). Not limited to COVID-19, integrative medicine including modern medicine, Yoga, mindfulness techniques, Ayurveda, and many more can be helpful for managing health and quality of life, and have the ability to reduce severity of disease. However, randomized trials are required to develop integrative healthcare.

It has been shown that meditation can potentially decrease the risk of acquiring cold and flu by improving physiological function and quality of life (40). Yogic breathing techniques improve respiratory and cardiac function, rendering it an effective tool to combat COVID-19 (41). Yoga will help calm down the mind and enhance immunity (17–20). PTSD due to natural disasters, epidemics, and wars has been shown to regress after practicing Yoga (42). Its application in the current pandemic using the digital module of Yoga and mindfulness may be helpful. If administered to COVID-19 patients under supervision, it may even reduce psychological stress (43). A few randomized controlled trials are taken up in the present pandemic that are investigating the efficacy of various breathing techniques including Yogic breathing in COVID-19 (44–46).

It is pertinent to note that we had stratified the Yoga group into a consolidated Yoga group, only *Asana*, only *Pranayama*, and only meditation groups. Meditation seemed to evoke beneficial outcomes than those practicing all, i.e., *Asana, Pranayamas*, and meditation. However, a longitudinal randomized trial is imperative to establish evidence. Our observation that the younger population preferred *Asanas* and *Pranayama*, while meditation was mainly preferred by the elderly, can help deliver innovative yoga protocols that make meditation more attractive to the young population and, consequently, useful for mental hygiene if presented as integrated yoga protocols (available at www.svyasa.edu.in). Our report highlights that a large number of subjects practiced consolidated Yoga (including *Asanas, Pranayama*, and meditation), proving the acceptability of an integrated module for school and college teachers. This could partly be due to the widespread popularity of both the Common Yoga Protocol released by the Ministry of AYUSH on the eve of International Day of Yoga, celebrated on June 21, as well as the widely published efficacy of Diabetic Yoga Protocol and COVID-19 Yoga Protocols released by the S-VYASA University (47, 48). These protocols include *Asana, Pranayama*, and meditation.

## Limitations of the Study

The study is limited by the fact that the sampling does not generate a study group that represents the general population as it was collected through social media. Second, the duration and regularity of Yoga practice before the lockdown was not assessed in the Yoga group, which would have given details of practice before lockdown. The same was assessed for lockdown period. Third, a proportionately higher number of younger subjects were found represented in the non-Yoga group; this might have influenced the outcome in a few components of the scales like the use of the Internet, physical strength, and others. Fourth, the self-reported COVID-19 symptoms could not be verified and RT-PCR for COVID-19 was not performed to establish increased susceptibility of non-Yoga group because of the lockdown restrictions. Fifth, a non-standard questionnaire was used. Mental health was not evaluated by any neuropsychological questionnaire such as the Perceived Stress Scale, but was based on the responses of CHAS.

We also could not rule out the role of physical exercises in improving mental and physical health; however, outdoor activities were restricted due to the lockdown. Furthermore, whether the restrictions mentioned earlier provided the environment conducive for the practice of *Asanas, Pranayamas*, and meditation, available through digital media, cannot be ascertained unless another validation study is carried out. In addition, we did not explore means to rule out repetitive form filling by the same individual due to error or intention; however, there were no rewards or benefits attached to completing the survey. Because the survey was administered online, the possibility of cognitive bias in the study was minimal; however, we entirely relied on the answers provided online without verifying the IP address, consistent with similar reports published elsewhere.

## Data Availability Statement

The raw data supporting the conclusions of this article will be made available by the authors, upon eligible request.

## Ethics Statement

The studies involving human participants were reviewed and approved by Swami Vivekananda Yoga Anusandhana Samsthana. Written informed consent for participation was not required for this study in accordance with the national legislation and the institutional requirements.

## Author Contributions

RN conceptualized the study, supervised data collection, and involved in discussions during article preparation. AA conceptualized the article. MR and MSS wrote and edited the article and contributed in data presentation. MNKS conceptualized the study. RK and JI performed the statistical analyses. AS created the scale and administered it nationally. HN envisioned the study, and inspired and guided the study to its completion imparting quality assurance. RN, AA, and VS critically reviewed and edited the article. All authors contributed to the article and approved the submitted version.

## Conflict of Interest

The authors declare that the research was conducted in the absence of any commercial or financial relationships that could be construed as a potential conflict of interest.

## Abbreviations

CHAS: COVID Health Assessment Scale
COVID-19: Coronavirus disease 2019
OR: Odds ratio
PTSD: Post-traumatic stress disorders
S-VYASA: Swami Vivekananda Yoga AnusandhanaSamsthana.
